# Mitochondrial Quality Control in Cardiomyocytes: A Critical Role in the Progression of Cardiovascular Diseases

**DOI:** 10.3389/fphys.2020.00252

**Published:** 2020-03-27

**Authors:** Hualin Fan, Zhengjie He, Haofeng Huang, Haixia Zhuang, Hao Liu, Xiao Liu, Sijun Yang, Pengcheng He, Huan Yang, Du Feng

**Affiliations:** ^1^Guangdong Provincial People’s Hospital, School of Medicine, South China University of Technology, Guangzhou, China; ^2^Guangzhou Municipal and Guangdong Provincial Key Laboratory of Protein Modification and Degradation, State Key Laboratory of Respiratory Disease, School of Basic Medical Sciences, Guangzhou Medical University, Guangzhou, China; ^3^Institute of Neurology, Affiliated Hospital of Guangdong Medical University, Zhanjiang, China; ^4^ABSL-Laboratory at the Center for Animal Experiment and Institute of Animal Model for Human Disease, Wuhan University School of Medicine, Wuhan, China; ^5^Department of Cardiology, Guangdong Cardiovascular Institute, Guangdong Provincial Key Laboratory of Coronary Heart Disease Prevention, Guangdong Provincial People’s Hospital, Guangdong Academy of Medical Sciences, Guangzhou, China; ^6^Department of Pulmonary and Critical Care Medicine, Hunan Provincial People’s Hospital, The First Affiliated Hospital of Hunan Normal University, Changsha, China; ^7^The Sixth Affiliated Hospital of Guangzhou Medical University, Qingyuan People’s Hospital, Guangzhou Medical University, Guangzhou, China

**Keywords:** mitochondrial quality control, mitophagy, post-translational modification, fission, fussion, cardiovascular disease, cardiomyocytes, cardiomyopathy

## Abstract

Mitochondria serve as an energy plant and participate in a variety of signaling pathways to regulate cellular metabolism, survival and immunity. Mitochondrial dysfunction, in particular in cardiomyocytes, is associated with the development and progression of cardiovascular disease, resulting in heart failure, cardiomyopathy, and cardiac ischemia/reperfusion injury. Therefore, mitochondrial quality control processes, including post-translational modifications of mitochondrial proteins, mitochondrial dynamics, mitophagy, and formation of mitochondrial-driven vesicles, play a critical role in maintenance of mitochondrial and even cellular homeostasis in physiological or pathological conditions. Accumulating evidence suggests that mitochondrial quality control in cardiomyocytes is able to improve cardiac function, rescue dying cardiomyocytes, and prevent the deterioration of cardiovascular disease upon external environmental stress. In this review, we discuss recent progress in understanding mitochondrial quality control in cardiomyocytes. We also evaluate potential targets to prevent or treat cardiovascular diseases, and highlight future research directions which will help uncover additional mechanisms underlying mitochondrial homeostasis in cardiomyocytes.

## Introduction

Mitochondria are double-membraned organelles which play important roles in cellular homeostasis, including producing energy by oxidative phosphorylation, maintaining calcium homeostasis, and regulating the signaling that leads to programmed cell death (Giorgi et al., 2018; Sprenger and Langer, 2019). The mitochondrion is composed of the outer mitochondrial membrane (OMM), the intermembrane space, the inner mitochondrial membrane (IMM) which is folded into projections called cristae, and the matrix. These substructures and compartments contain a number of proteins which maintain mitochondrial metabolism and homeostasis (Hammerling and Gustafsson, 2014). However, under stresses from environmental insults, mitochondrial shape, membrane potential and metabolism will be disturbed, and mitochondrial DNA, reactive oxidative species (ROS) and cytochrome c may be released into the cytoplasm. Moreover, mitochondrial proteins may even misfold under more severe conditions, causing a plethora of diseases such as cardiovascular disease (CVD), neurological diseases, aging and so on (Anzell et al., 2018; Tahrir et al., 2019).

Mitochondrial quality control (mitochondrial QC) pathways include post-translational modification (PTM) of mitochondrial proteins, mitochondrial dynamics (biogenesis, fission, and fusion) and mitochondrial autophagy (mitophagy). Together, these pathways repair or remove dysfunctional and damaged mitochondria to maintain mitochondrial morphology, quantity, and function, as well as to promote cell survival (Suliman and Piantadosi, 2015).

**FIGURE 1:**
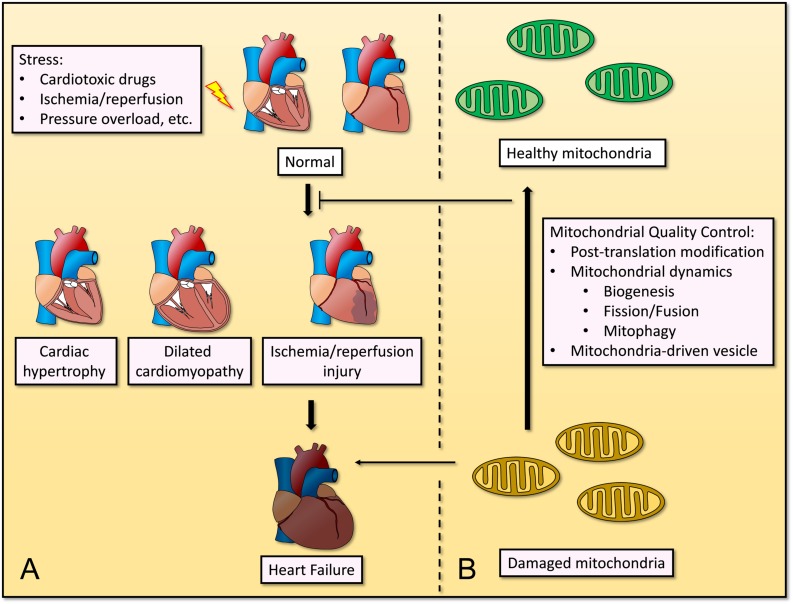
Mitochondrial quality control in cardiovascular disease under stress. **(A)** Stresses such as cardiotoxic drugs, ischemia/reperfusion, pressure overload, etc., causes progression of several cardiovascular diseases, including cardiac hypertrophy, dilated cardiomyopathy, ischemia/reperfusion injury, and even heart failure. **(B)** Mitochondrial quality control including post-translation modification, mitochondrial dynamics (biogenesis, fission/fusion, and mitophagy) and mitochondria-driven vesicle, restores mitochondrial homeostasis and inhibits the progression of cardiovascular diseases.

Cardiovascular disease is the one of most common causes of death globally, and its incidence and prevalence have increased over the years (Kyu et al., 2018). Remodeling of cardiomyocytes occurs in heart failure, ischemia/reperfusion (I/R) injury, and hypertrophic or dilated cardiomyopathy, which are characterized by mitochondrial dysfunction and perturbed cellular homeostasis (Tahrir et al., 2019). Given the high energy demands of cardiomyocytes as they pump blood around the body under normal physiological conditions, deregulated mitochondrial homeostasis causes cardiac dysfunction and remodeling, and will further induce CVD and complications. Through mitochondrial QC, the abnormal function and structure of heart will be improved (Figure 1).

In this review, we will discuss the progress in searching for evidence of the role of mitochondrial QC in cardiomyocytes, and for understanding the association between mitochondrial QC and development of CVD such as ischemia/reperfusion injury, cardiomyopathies, etc.

## The Molecular Mechanisms of Mitochondrial Quality Control (QC)

### Post-translational Modification (PTM) of Mitochondrial Proteins

In physiological and pathological conditions, the activity of mitochondrial proteins is regulated by PTMs, including SUMOylation, ubiquitination, phosphorylation, acetylation and so on, to maintain mitochondrial metabolism or to trigger mitochondrial fission/fusion and mitophagy (Klimova et al., 2018).

#### SUMOylation

SUMOylation is a kind of PTM in which Small Ubiquitin-like Modifier (or SUMO) proteins are covalently attached to lysine residues of the substrate protein by SUMO E3 ligase. SUMO-1, SUMO-2, and SUMO-3 have been identified as the main members of the SUMO family (Hendriks and Vertegaal, 2016). SUMO is translated as an inactive precursor and subsequently cleaved by proteases such as the Sentrin/small ubiquitin-like modifier-specific protease (SENP) family to produce the mature form. SUMO undergoes an ATP-dependent interaction with SUMO E1 enzyme to form an E1-SUMO thioester conjugate through sequential adenylation and thioester bond formation at the carboxyl terminus of SUMO. Subsequently, SUMO is transferred to a cysteine residue of the SUMO E2 conjugating enzyme to form an E2-SUMO thioester bond, which then interacts with the E3 enzyme to facilitate transfer of SUMO to the substrate (Gareau and Lima, 2010; Wilkinson and Henley, 2010).

Several previous studies have reported that mitochondrial proteins are post-translationally modified via SUMOylation. Mitochondrial-anchored protein ligase (MAPL), the first known mitochondrial-anchored SUMO E3 ligase, can SUMOylate Dynamin relative-protein 1 (DRP1) and induce mitochondrial fission, but the mechanism was not clear (McBride et al., 2009). Further studies revealed that DRP1, is SUMOylated via MAPL-dependent activity of Bax/Bak during programmed cell death. SUMOylated DRP1 stabilizes an ER/mitochondrial signaling platform required for mitochondrial constriction, calcium flux, crista remodeling, and efficient cytochrome c release (Wasiak et al., 2007; Prudent et al., 2015). SENP3 is degraded during oxygen/glucose deprivation (OGD) and brain ischemia. This reduces deSUMOylation of DRP1 and leads to cytochrome c release and programmed cell death (Guo et al., 2013). Interestingly, as well as deSUMOylating DRP1, SENP3 also promotes the binding of DRP1 to mitochondrial fission factor (MFF), which leads to mitochondrial fragmentation and cytochrome c release (Guo et al., 2017).

#### Ubiquitination

Ubiquitination is a post-translational modification process that is critical for protein or organelle degradation. Ubiquitin is covalently attached to lysine residues on substrate proteins via Ub-activating enzymes (E1), Ub-conjugating enzymes (E2), and Ub-ligase enzymes (E3). Ub is activated by E1, and is subsequently transferred to E2 to form Ub-E2. The Ub-E2 conjugate then interacts with E3 and the substrate, which results in formation of an isopeptide bond between the C-terminus of Ub and a lysine residue in the substrate (Wang L. et al., 2019). Successive ubiquitination reactions between one ubiquitin to another lead to formation of a polyubiquitin chain, which binds to the proteasome and leads to degradation of the substrate (Ciechanover, 2005).

Several mitochondrial proteins are degraded via the Ubiquitination Proteasome System (UPS) pathway to maintain cellular homeostasis. The gene encoding Parkin, which serves as a Ub-ligase enzyme, is mutated in Parkinson’s disease (Kitada et al., 1998). DRP1 interacts with Parkin and is subsequently ubiquitinated by Parkin for degradation. The depletion of Parkin reduces the ubiquitination and degradation of DRP1, causing increased mitochondrial fission (Wang et al., 2011; Buhlman et al., 2014). Ubiquitination of DRP1 is also catalyzed by APC/CCdh1 E3 ubiquitin ligase complex, leading to changes in mitochondrial dynamics when cellular mitosis is completed (Horn et al., 2011). MARCH-V, a ubiquitin ligase, promotes the formation of elongated mitochondria by ubiquitinating DRP1 and interacting with mitofusin (MFN) 2 (Nakamura et al., 2006). Upon inhibition of USP30 by the diterpenoid derivative 15-oxospiramilactone (S3), non-degradative ubiquitination of MFN1/2 is induced to promote mitochondrial fusion (Yue et al., 2014). Additionally, ubiquitination also regulates mitophagy. For example, in cells treated with CCCP, which induces loss of mitochondrial membrane potential, MFN2 is ubiquitinated by the E3 ubiquitin ligase Parkin for degradation, which leads to impaired mitochondrial fusion and mitophagy (Gegg et al., 2010; Poole et al., 2010; Tanaka et al., 2010; Glauser et al., 2011). Also, MFN2 is ubiquitinated by the PINK/Parkin pathway to trigger p97-dependent disassembly of MFN2 complexes, which disrupts the contacts between mitochondria and ER and subsequent initiation of mitophagy (Tanaka et al., 2010; McLelland et al., 2018). Furthermore, the ubiquitination of other mitochondrial proteins such as MID49, MCL1 and FIS1 plays a role in mitochondrial dynamics and cellular homeostasis (Zhang et al., 2012; Cherok et al., 2017).

#### Phosphorylation

Phosphorylation is a typical form of PTM which occurs at several sites of target proteins such as serine, threonine, or tyrosine residues. DRP1 has been most widely studied to elucidate the mechanism of phosphorylation-mediated mitochondrial fragmentation. Phosphorylation of DRP1 occurs on several sites, such as Ser616 or Ser637 in human, and is catalyzed by several kinases, including PKA (Chang and Blackstone, 2007), CaMK1α (Cribbs and Strack, 2007; Han et al., 2008), CDK1, or PKδ (Zaja et al., 2014; Klimova et al., 2018). In addition, phosphorylation of DRP1 by c-Abl mediates oxidative stress-induced mitochondrial fragmentation and cell death (Chen and Dorn, 2013; Zhou H. et al., 2017). Under high-glucose conditions, a transient increase in Ca^2+^ levels and ROS production is induced, followed by activation of ERK1/2, which phosphorylates DRP1 and promotes mitochondrial fragmentation (Yu et al., 2011). In addition, other mitochondrial QC processes are mediated by phosphorylation of mitochondrial proteins. MFF is phosphorylated by adenosine monophosphate-activated protein kinase (AMPK) in response to energy stress, which is required for DRP1-mediated mitochondrial fission (Toyama et al., 2016). The pleiotropic ERK kinase phosphorylates MFN1 to reduce the mitochondrial fusion rate and to cause mitochondrial permeabilization (Pyakurel et al., 2015). By phosphorylating MFN2, PKA inhibits the proliferation of vascular smooth muscle cells, while PINK1 facilitates mitochondrial fragmentation and promotes mitophagy (Chen and Dorn, 2013; Zhou et al., 2010; Gong et al., 2015). FUNDC1, a mitophagy receptor under hypoxic conditions, is phosphorylated by ULK1 at Ser17. This enables FUNDC1 to bind to LC3, which leads to mitophagy for clearance of damaged mitochondria (Wu et al., 2014). Interestingly, under hypoxic conditions, Src kinase-mediated phosphorylation of FUNDC1 at Tyr18 in LIR motif is inhibited. This results in dephosphorylation of FUNDC1 and increased mitophagy (Liu et al., 2012).

#### Acetylation/Deacetylation

Acetylation is an evolutionarily conserved PTM, which plays an important role in gene expression, gene function and cellular metabolism via regulation of histones and non-histone proteins (Baeza et al., 2016). Recently, accumulating lines of studies have focused on lysine acetylation of non-histone proteins in mitochondria, which modulates mitochondrial homeostatic processes including mitochondrial dynamics and mitophagy. Initially, lysine acetylation was discovered as a PTM of histones which regulates the expression and function of nuclear genes and is mediated by histone acetyltransferases (HATs) and histone deacetylases (HDACs). Subsequently, lysine acetylation was identified to occur outside the nucleus. This form of lysine acetylation is mediated by lysine acetyltransferases (KATs) and lysine deacetylases (KDACs) (Drazic et al., 2016). The exact number of KATs is unclear, but all identified KATs can be divided into three families: the GNAT family, the MYST family and the p300/CBP family. The human genome encodes 18 KDACs, which can be divided into two types: Zn^2+^-dependent HDACs and NAD^+^-dependent sirtuin deacetylases (Takeo et al., 2019). Multiple associations between acetylation or deacetylation and diseases have been revealed, including neuronal degeneration (Min et al., 2015; Li Y. et al., 2018), cancer (Audia and Campbell, 2016), disorders of glucose homeostasis (Mihaylova et al., 2011; Winkler et al., 2012), and cardiomyopathy(Vakhrusheva et al., 2008).

### Mitochondrial Dynamics

Mitochondrial dynamics, including biogenesis, fusion, fission, and mitophagy, regulate mitochondrial morphology and maintain mitochondrial population health, which is critical for cellular responses to metabolic cues or environmental stresses. In physiological conditions, mitochondria continually and spontaneously produce reactive oxygen species, accompanied by the production of ATP, which impairs proteins, lipids and DNA within mitochondria. To eliminate these deleterious components and to maintain mitochondrial metabolism, fission helps the mitochondrion to segregate the deleterious components into a daughter mitochondrion which will be degraded by mitophagy. Mitochondrial fusion is necessary for maintaining the capacity of oxidative phosphorylation by healthy ones fused with damaged ones to compensate the deficient metabolism (Youle and van der Bliek, 2012).

Mitochondrial dynamics are mediated by several factors. In brief, the transcriptional coregulator peroxisome proliferator-activated receptor γ (PPARγ) coactivator 1α (PGC1α) is the core in mitochondrial biogenesis (Dorn et al., 2015); MFF, FIS1, MID49, and MID51 are required to recruit the fission factor DRP1 during mitochondrial fission, and OPA1 and MFN1/2 are required for mitochondrial fusion; characterized by PINK1/Parkin pathway, multiple pathways involve in mitophagy for the degradation of senescent and damaged mitochondria (Youle and van der Bliek, 2012).

#### Mitochondrial Biogenesis

PGC1 plays a key role in mitochondrial biogenesis through activating PPAR, increasing content of mitochondrial DNA (mtDNA), and promoting expression of UCP1 and key mitochondrial enzymes of the respiratory chain in brown fat and skeletal muscle (Puigserver et al., 1998). PGC1 is regulated by several factors, such as cAMP, AMPK, and SIRT1, and plays its core role in mitochondrial biogenesis (Wu et al., 1999; Gerhart-Hines et al., 2007; Little et al., 2010). Through interacting with transcription factors at downstream, including NRF1/2, ERR and PPAR, PGC1 regulate the replication of mtDNA and the transcription of mitochondrial proteins genes for mitochondrial biogenesis (Dorn et al., 2015).

#### Mitochondrial Fission and Fusion

For mitochondrial fission, DRP1 is recruited from the cytosol to form spiral oligomers around the mitochondrion. DRP1 binds to 4 protein receptors, MFF, FIS1, MID49, and MID51, that constrict the mitochondrion and divide it into two daughters, in which mitochondrial components are asymmetrically distributed or segregated (Dorn and Kitsis, 2015; Ni et al., 2015). By fission, a daughter mitochondrion which may contain deleterious or damaged components can be removed via mitophagy (Youle and van der Bliek, 2012). The site where DRP1 constricts mitochondria is often the same place where mitochondria contact the ER (Friedman et al., 2011). In addition, fission is modulated via PTM of DRP1 as mentioned above. These PTMs, including SUMOylation and phosphorylation, are essential for the maintenance of mitochondrial homeostasis.

Mitochondrial fusion in mammals is mediated by MFN1 and MFN2, two membrane-anchored dynamin-related GTPases, which regulate fusion of the outer mitochondrial membrane, and by OPA1, also a dynamin-related GTPase, which regulates fusion of the IMM (Youle and van der Bliek, 2012; Ni et al., 2015).

### Mitophagy

Autophagy is an evolutionarily conserved pathway in which double-membraned structures called autophagosomes encapsulate damaged organelles, protein aggregates, and invading microorganisms and fuse with lysosomes for degradation (Levine and Kroemer, 2008; Martinet and De Meyer, 2009; Gatica et al., 2015; Ikeda et al., 2015a). Macroautophagy is widely studied and is considered as the major cellular pathway for non-selective degradation of proteins and organelles. In addition to non-selective macroautophagy, selective autophagy, which acts via dedicated receptors on autophagic substrates, plays an important role in removal of specific organelles such as mitochondria (mitophagy), the ER (reticulophagy), ribosomes (ribophagy), lysosomes (lysophagy), etc. (Galluzzi et al., 2017).

Here, we focus on mitophagy, a critical mitochondrial QC process for maintenance of mitochondrial homeostasis, and we examine the link between mitophagy and physiological and pathological changes in cardiomyocytes.

Damaged mitochondria are removed by mitophagy via the PINK1-Parkin axis, which is the most widely studied mitophagy pathway. In normal conditions, after being imported into the IMM, PINK1 is constitutively cleaved by the IMM protease PARL, and is then released to the cytoplasm for further degradation. Upon loss of mitochondrial membrane potential, the import of PINK1 is blocked and therefore PINK1 cannot be accessed by PARL, resulting in its stabilization on the OMM. Subsequently, PINK1 phosphorylates Parkin and ubiquitin and further induces mitophagy to remove damaged mitochondria (Youle and van der Bliek, 2012; Lazarou et al., 2015; Nguyen et al., 2016; Pickles et al., 2018).

In addition to PINK1 and Parkin, other LC3-interacting proteins such as the mitophagy receptors FUNDC1, BNIP3, or BNIP3L/NIX are also involved in mitophagy (Liu et al., 2014).

### Mitochondrial-Derived Vesicles (MDVs)

In addition to PTMs on mitochondrial proteins, changes in mitochondrial dynamics and mitophagy, increasing evidence has indicated that mitochondrial-derived vesicles (MDVs) are important for mitochondrial QC. In response to different stimuli, like starvation, small vesicles carrying specific components of mitochondria are generated and transported to lysosomes for degradation via the PINK1/Parkin pathway to maintain cellular homeostasis (Soubannier et al., 2012; McLelland et al., 2014; Suliman and Piantadosi, 2015). The size of MDVs ranges from 70 to 150 nm and the generation of MDVs is independent of DRP1 (Sugiura et al., 2014). Altogether, there are three distinct criteria to identify MDVs: independence from the core fission GTPase DRP1, incorporation of selected mitochondrial cargo, and a diameter of 70–150 nm as observed by electron microscopy (Cadete et al., 2016).

In summary, mitochondrial QC exerts its function to maintain mitochondrial and cellular homeostasis via multiple components, which is interacting with each other. PTMs serve as triggers to keep the balance of mitochondrial dynamics and MDVs. The deficiency or excess of PTMs is associated with abnormal mitochondrial dynamics and MDVs, which is involved in several pathological progressions in CVD.

## The Mitochondrial Quality Control (QC) in Cardiovascular Disease (CVD)

### Post-translational Modification (PTM) of Mitochondrial Proteins in Cardiomyocytes

#### SUMOylation

DJ-1, also known as Parkinson disease protein 7, serves as a molecular chaperone and is activated in oxidative environments. Mutations in the gene encoding DJ-1 are associated with Parkinson’s disease (Shendelman et al., 2004). Deficient DJ-1 enhanced accumulation of SUMO-1 modified proteins, reduced SUMO-2/3 modified proteins after I/R injury, which is associated with enhanced SUMOylation of DRP1 and excessive mitochondrial fission. The DJ-1 deletion heart displayed increased infarction size, deteriorated cardiac function *in vivo*. That study suggested that the activation of DJ-1 attenuates excessive mitochondrial fission and protects cardiomyocytes from I/R injury by decreasing the SUMOylation of DRP1 (Shimizu et al., 2016). In contrast, another study suggests that deSUMOylation of DRP1 is involved in the release or cleavage of apoptotic factors, impaired mitochondrial fission, and development of cardiomyopathy induced by overexpression of SENP5 *in vivo* (Kim et al., 2014). Treatment with zinc protects cardiomyocytes from hypoxia/reoxygenation (H/R) injury *in vitro* and improves cardiac function against I/R injury *ex vivo* by inducing the SUMOylation of DRP1 and promoting mitophagy (Bian et al., 2019).

In summary, SUMOylation of DRP1 mediates mitochondrial dynamics and mitophagy, but the exact mechanism remains elusive. The existing research appears to suggest the levels of DRP1 SUMOylation should be controlled in a restrict range to maintain cardiac function. The association between SUMOylation of other mitochondrial proteins and cardiac protection remains unclear and needs further investigation.

#### Ubiquitination

In cardiomyocytes, beside mitophagy mediated by mitochondrial ubiquitination, ubiquitination regulates the cardiomyocytes homeostasis through specific function of protein substrates via activated UPS. Mitochondrial Rho GTPase 2 (Miro2) is an OMM protein which facilitates the formation of mitochondrial nanotubes along microtubules and mediates the transportation of mitochondria. The expression of Parkin increases during cardiac hypertrophy, which leads to ubiquitination of Miro2 and causes impairment of mitochondrial communication, whereas overexpressed Miro2 protects cardiomyocytes from transverse aortic constriction-induced cardiac dysfunction *in vivo* (Cao et al., 2019). On the contrary, VDAC1, a substrate of Parkin, is ubiquitinated when HL-1 mouse atrial cardiomyocytes are exposed to arsenic trioxide (ATO). This maintains mitochondrial homeostasis and protects cardiomyocytes during exposure to cardiotoxic agents *in vitro* (Watanabe et al., 2014).

In summary, previous studies have shown that ubiquitination of several mitochondrial proteins by PINK/Parkin or MARCH-V triggers changes of mitochondrial dynamics and mitophagy to maintain cellular homeostasis. However, the mechanism by which ubiquitination mediates mitochondrial QC as well as the different functions with specific ubiquitination of substrates in cardiomyocytes remains unclear and should be further investigated *in vivo* and *in vitro*.

#### Phosphorylation

Recently, several studies have focused on PTMs of mitochondrial proteins via phosphorylation. Fasudil has been reported to reduce mitochondrial fragmentation by inhibiting the effect of the RhoA/ROCK pathway, which catalyzes phosphorylation of DRP1, and subsequently induces mitophagy and protects cardiomyocytes from endotoxemia-induced impairment of heart function *in vivo* (Preau et al., 2016). Interestingly, in another study, RhoA activation was suggested to regulate the phosphorylation of DRP1 for cardioprotection *in vitro* (Brand et al., 2018). Furthermore, a recent study reported that protein kinase D (PKD), a downstream kinase of Gq protein-coupled receptor (GqPCR) signaling, phosphorylates DRP1 and promotes mitochondrial fragmentation in cardiomyocytes via α1-AR-PKD signaling *in vitro* and *in vivo* (Jhun et al., 2018). The protective effect of this pathway on stressed cardiac cells and the underlying mechanism should be further investigated. Also, MFN2 is a well-studied target protein phosphorylated by PINK1 for mitochondrial QC in cardiomyocytes (Chen and Dorn, 2013), which also plays a critical role in perinatal cardiac maturation (Gong et al., 2015). In addition, there is evidence that AMPK-α2 will be switched to AMPK-α1 increasingly in heart failure, which reduces the level of AMPK-α2 and promotes the development of cardiac dysfunction, whereas AMPK-α2 interacts with phosphorylated PINK1 to initiate PINK/Parkin-mediated mitophagy for protection against progression of heart failure under phenylephrine stimulation *in vivo* (Wang B. et al., 2018).

Like ubiquitination (discussed above), phosphorylation plays an important role in QC of mitochondria, and also triggers changes of mitochondrial dynamics and mitophagy. However, the association between the PTM and cardiomyocyte function in physiological and pathological processes is clearer for phosphorylation than for ubiquitination. Nevertheless, the functions of phosphorylation in heart cells still need to be further investigated.

#### Acetylation/Deacetylation

Several studies have investigated the link between the lysine acetylation/deacetylation balance and mitochondrial homeostasis in cardiomyocytes to understand the progress of CVDs. OPA1 is hyperacetylated under pathological stress and its GTPase activity is suppressed, whereas SIRT3, a NAD-dependent protein deacetylase (Onyango et al., 2002), can deacetylate OPA1 at Lys926 and Lys931 and restore its GTPase activity *in vivo* (Samant et al., 2014). These changes are accompanied by altered mitochondrial cAMP/PKA signaling (Signorile et al., 2017). Additionally, acetylation participates in regulation of mitophagy. SIRT3 depletion facilitates angiotensin II-induced PINK/Parkin acetylation and increases ROS generation, leading to impaired mitophagy, while the overexpression of SIRT3 enhances PINK/Parkin-dependent mitophagy, reduces ROS generation, restores vessel sprouting and tube formation, and improves cardiac function and microvascular network formation *in vivo* (Wei et al., 2017). Similarly, SIRT3 catalyzes the acetylation of Forkhead box protein O (FOXO) 3, which is upstream of Parkin-dependent mitophagy (Celestini et al., 2018), to maintain mitochondrial homeostasis during streptozotocin-induced diabetic cardiomyopathy *in vivo* (Das et al., 2014). In addition, deacetylation has been reported to regulate the levels of mitochondrial metabolism proteins that localize in the mitochondrial matrix, such as superoxide dismutase (SOD) 2 (Dikalova et al., 2017), nicotinamide mononucleotide adenylyltransferase (NMNAT) 3 (Yue et al., 2016), and glycogen synthase kinase (GSK) 3β (Sundaresan et al., 2015), to protect cardiomyocytes from hypertension, hypertrophy, and cardiac fibrosis *in vivo*. Furthermore, there is evidence that SIRT4 promotes development of hypertension by inhibiting the binding of manganese superoxide dismutase (MnSOD) to SIRT3. This results in decreased deacetylation of MnSOD by SIRT3 and reduced activity of MnSOD, and ROS accumulation during angiotensin II treatment *in vivo* (Luo et al., 2017).

Accumulating evidence reveals that the balance between acetylation and deacetylation has a crucial role in mitochondrial dynamics, mitophagy and mitochondrial metabolism in cardiomyocytes. These mitochondrial QC processes prevent the progression of CVD. Further research is needed to find effective pharmacological agents that target the sites of acetylation, which may lead to the development of new therapies for CVD.

**FIGURE 2:**
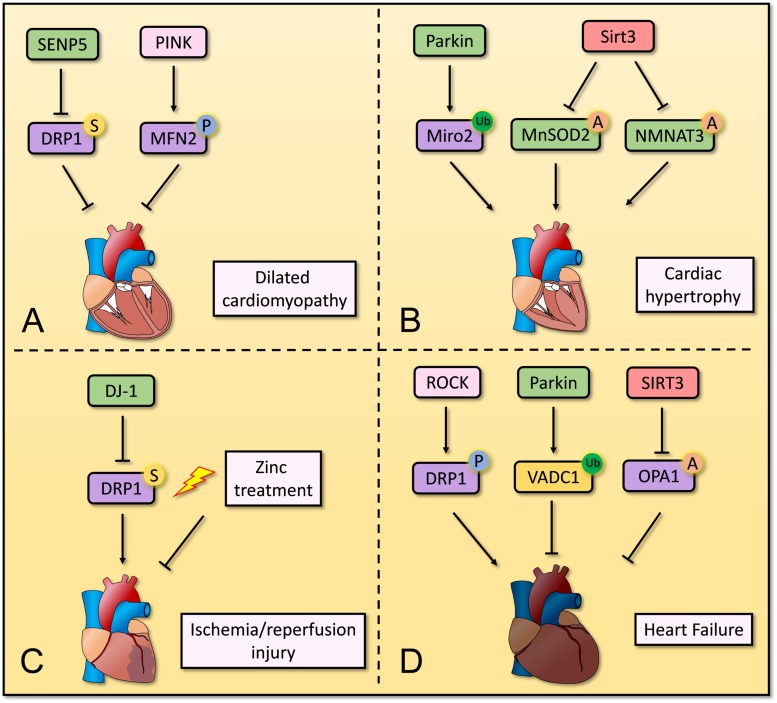
Overview of post-translation modification in mitochondria in cardiomyocytes. Several post-translation modifications of mitochondrial proteins play important roles in mitochondrial homeostasis in cardiomyocytes. **(A)** Decreased SUMOylation of DRP1 and increased SUMOylation of MFN2 repress the progression of dilated cardiomyopathy. **(B)** Parkin induces ubiquitination of Miro2 and its degradation, leading to development of cardiac hypertrophy. SIRT3 deacetylates MnSOD2 and NMNAT3 to restores normal cardiac function and structure. **(C)** DJ-1 and Zinc treatment contribute to suppressing ischemia/reperfusion injury by regulating SUMOylation of DRP1. **(D)** Phosphorylation of DRP1 via RhoA/ROCK pathway induces heart failure, whereas ubiquitination of VADC1 and deacetylation of OPA1 repress development of heart failure.

PTMs are crucial for mitochondrial homeostasis in cardiomyocytes, and dysregulation of PTMs is associated with development of CVD. Although different kinds of PTMs have been reported for mitochondrial proteins, further mechanistic studies in cardiomyocytes are needed (Figure 2).

## Mitochondrial Dynamics in Cardiovascular Disease (CVD)

For maintaining the pump function to meet the demand of organism, cardiomyocytes require health mitochondrial homeostasis to provide sufficient ATP for energy. Since mitochondria cannot be synthesized *de novo*, the dynamical renew cycle, including mitochondrial biogenesis, fission, fusion, and mitophagy, is required to maintain mitochondrial homeostasis health in cardiomyocytes (Suliman and Piantadosi, 2015). At baseline or in unstressed conditions, the expression of mitochondrial fusion-related proteins is higher than that of fission-related and mitophagy-related proteins in adult mouse heart (Song et al., 2015a). The high levels of fusion may contribute to maintaining the capacity of oxidative phosphorylation (Youle and van der Bliek, 2012), which meets the demand of cardiomyocytes.

### Mitochondrial Biogenesis in Cardiomyocytes

Lehman et al. (2000) firstly reported the role of PGC1 in mitochondrial biogenesis in cardiomyocytes. The overexpression of PGC1 induced upregulated expression of mitochondrial proteins, accumulation of enlarged mitochondria *in vitro* and resulted in the loss of sarcomeric structure and a dilated cardiomyopathy *in vivo* (Lehman et al., 2000). Deficient PGC1 was not associated with abnormal mitochondrial and cardiac structure, although it caused the deficient expression of mitochondrial proteins and cardiac contractile dysfunction in physiological conditions *in vivo* (Arany et al., 2005). The same group further revealed that when PGC1 deficiency mice were subjected to transverse aortic constriction, their heart appeared to enlarge and be heavier, consistent with hypertrophy *in vivo* (Arany et al., 2006).

In addition to PGC1, other factors also were reported to participate in the regulation of mitochondrial biogenesis. Heme oxygenase (HO) 1, a protective antioxidant enzyme that prevents cardiomyocyte apoptosis, promotes the endogenous generation of carbon monoxide (CO), which increases NRF1 levels via AKT-GSK3β-NRF2 pathways. Consequently, the overexpression of HO1 causes the accumulation of NRF1 to increase the mtDNA replication and promotes mitochondrial biogenesis, leading to protecting cardiomyocytes from cardiotoxic stress *in vivo* (Piantadosi et al., 2008).

These studies indicated that mitochondrial function is redundant to the demand for energy in cardiomyocytes, while mitochondrial biogenesis plays a crucial role when cardiomyocytes are subjected to external environmental stress, accompanied by increasing of mitophagy.

#### Mitochondrial Fission in Cardiomyocytes

In cardiomyocytes, exogenous inhibition of DRP1 improves impaired cardiac function, whereas endogenous DRP1 knockout promotes cardiac dysfunction. Reduced DRP1 expression levels induced by Mdivi-1, a mitochondrial fission inhibitor, reduced cell death, inhibited mitochondrial permeability transition pore (mPTP) opening, and protected cardiomyocytes from I/R injury *in vitro*. The pretreatment of Mdivi-1 followed by I/R injury improved LV developed pressure, reduced LV end-diastolic pressure and myocardial infarction size *in vivo* and *ex vivo* (Ong et al., 2010; Sharp et al., 2014). Besides Mdivi-1, the pretreatment of FK506 and therapeutic hypothermia (30°C) also preserved cardiac function via preventing Drp1-S637 dephosphorylation (Sharp et al., 2014).

Cardiac-specific DRP1 knockout mice have suppressed autophagy, dysfunctional mitochondria, and greater infarct size after ischemia/reperfusion. They also develop left ventricular dysfunction, and die after 13 weeks. *In vitro*, accumulated enlarged mitochondria induced by impaired mitochondrial fission fail to be degraded in lysosomes, although recruits abundant p62 and ubiquitin (Ikeda et al., 2015b). Interestingly, concomitant conditional deletion of Parkin suppresses the cardiac-specific DRP1 knockout-induced impaired ventricular ejection, adverse cardiac remodeling, and cardiac myocyte necrosis and fibrosis, but does not alter mitochondrial enlargement in cardiomyocytes (Song et al., 2015a). On the contrary, the whole body Parkin knockout mice fail to improve the function of heart with specific knockout of DRP1 (Kageyama et al., 2014). The underlying mechanism may be explained by the lower recruitment levels of p62 and ubiquitin on mitochondria in the former, which appears to block the degradation of mitochondria and maintain the cardiac function to some extent. The potential mechanisms should be also investigated in further study. These studies suggest that mitochondrial fission regulates cardiac function via mitophagy in physiological conditions.

Recently, it was reported that the interaction between DRP1 and FIS1 is enhanced under LPS treatment, and this is associated with increased ROS generation, mitochondrial fragmentation, and increased mortality *in vitro*. These effects are attenuated by treatment with P110 on LPS-treated mice, which inhibits the interaction between DRP1 and FIS1 (Haileselassie et al., 2019). Evidence also showed that cardiac dysfunction in Huntington’s disease is related to DRP1/FIS1-mediated impairment of lysosomal function. Abnormal mitochondrial fragmentation and dysfunctional lysosomes were observed in H9C2 embryonic cardiomyocytes expressing mutant Huntingtin protein with a long polyglutamine repeat (Q73) and in human iPSC-derived cardiomyocytes transfected with Q77 (Joshi et al., 2019). In addition to DRP1, mitochondrial fission and fusion are also affected in phenylephrine (PE)-induced hypertrophy, where the mitochondrial permeability transition pore opens and the mitochondria are exposed to oxidative stress *in vivo* (Javadov et al., 2011).

#### Mitochondrial Fusion in Cardiomyocytes

Mitochondrial fusion has been reported to regulate the homeostasis of cardiomyocytes. Reduced expression of MFN2 and mitochondrial fission are observed in diabetic hearts from 12-week-old leptin receptor-deficient mice and in cardiomyocytes fed with high-glucose and high-fat medium. This is due to down-regulation of PPARα, which directly binds to the promoter of MFN2 and modulates its expression. The functional deficits in cardiomyocytes are attenuated by reconstitution of MFN2, which enhances mitochondrial fusion (Hu et al., 2019). The endogenous deficiency of MFN2 leads to hypertrophy and slight cardiac dysfunction, which is associated with a marked delay in mitochondrial permeability transition. Followed by the I/R procedure, MFN2 ablation results in improved cardiac performance and lower cell death ratio (Papanicolaou et al., 2011). In contrast, further research indicated that cardiac-specific MFN2 knockout mice caused a higher cell death ratio after the I/R procedure. MFN2 ablation in cardiomyocytes prevented the fusion between autophagosomes and lysosomes for mitochondrial degradation and caused the accumulation of autophagosomes (Zhao et al., 2012). These studies indicated the role of MFN2 in cardiomyocytes and cardiac function should be further investigated.

Optic atrophy 1 (OPA1) is also involved in mitochondrial homeostasis in cardiomyocytes. Reduction of OPA1 is associated with ischemia-induced heart failure, and with mitochondrial fragmentation and apoptosis in cardiomyocytes. Overexpression of OPA1 fails to prevent apoptosis, although it does increase the tubularity of mitochondria *in vitro* (Chen et al., 2009). However, further study *in vivo* suggested that overexpression of OPA1 refined cardiac injury accompanied by the upregulated long-OPA1 levels *in vivo*. The potential mechanism may be OPA1-dependent stabilization of mitochondrial cristae structure increases mitochondrial respiratory function, blocks cytochrome c release, ROS production, and apoptosis (Varanita et al., 2015).

In addition to MFN2 and OPA1, SIRT3 is another important factor that protects cardiomyocytes from post-infarction cardiac injury by normalizing the AMPK-DRP1 pathways. This has the effect of attenuating mitochondrial fission and preserving mitochondrial homeostasis, which improves cardiac fibrosis, maintains myocardial function, inhibits inflammatory responses, and reduces cellular death (Liu et al., 2019). Similar to SIRT3, NR4A1, which has no fission or fusion activity, promotes MFF-dependent mitochondrial fission and represses mitophagy, resulting in aggravation of cardiac microvascular I/R injury *in vivo* (Zhou H. et al., 2018). NR4A1 has also been reported to play an important role in protecting against vascular injury (Papac-Milicevic et al., 2012).

As mentioned above, Moshi Song and colleagues discovered that concomitant conditional Parkin deletion suppresses the effect of DRP1 ablation in heart (Ikeda et al., 2015b). To further investigate the association between mitochondrial dynamics and mitochondrial QC, the same group revealed that DRP1 conditional knockout in cardiomyocytes induces mitochondrial enlargement, dilated cardiomyopathy, and cardiomyocyte necrosis. Furthermore, MFN1/2 conditional knockout causes mitochondrial fragmentation and eccentric ventricular remodeling, namely eccentric hypertrophy without change in the ratio of left ventricular end-diastolic radius to wall thickness. Contrary to previous observations, DRP1 ablation increased mitophagy in this study, as evidenced by accumulation of LC3-II and increased engulfment of mitochondria into lysosomes (Song et al., 2015b).

**FIGURE 3:**
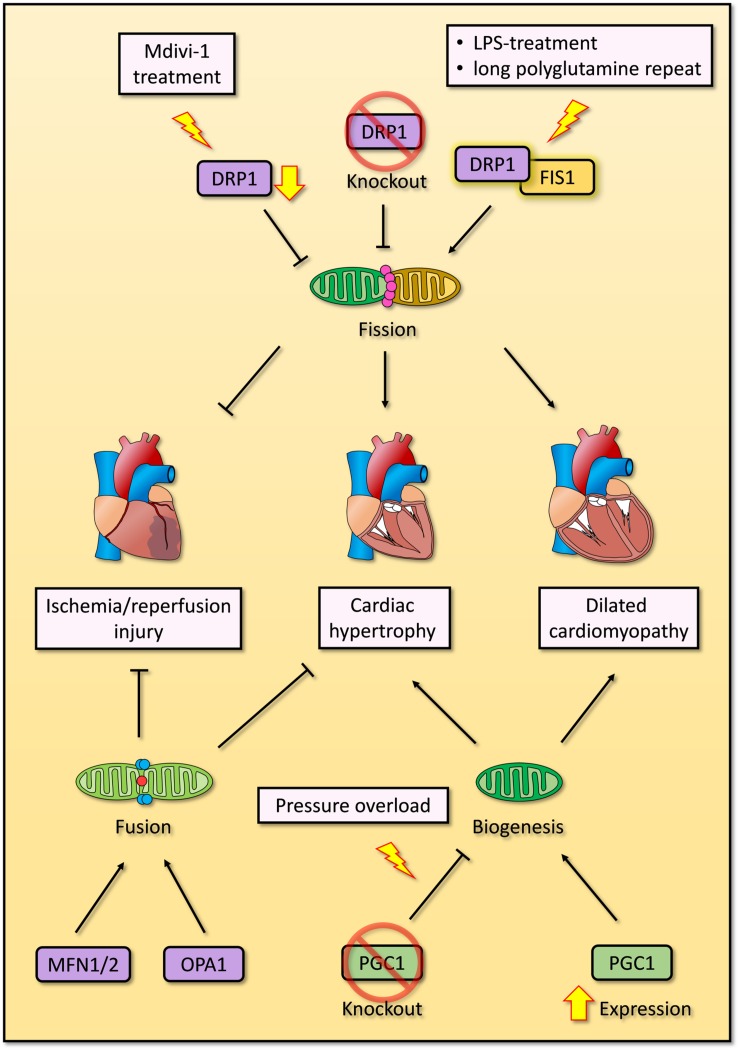
Overview of mitochondrial biogenesis, fission and fusion in cardiomyocytes. Overexpression of PGC1 induces mitochondrial biogenesis and dilated cardiomyopathy, while deficient PGC1 results in cardiac hypertrophy under pressure overloading. Exogenous inhibition of DRP1 by Mdivi-1 improves impaired cardiac function after ischemia/reperfusion injury, whereas endogenous DRP1 knockout promotes cardiac hypertrophy. Enhanced interaction between DRP1 and FIS1 induced by LPS treatment or expression of long polyglutamine repeat causes excessive mitochondrial fission and dilated cardiomyopathy. Two crucial GTPase proteins of mitochondrial fusion, MFN1/2 and OPA1, are essential to repress cardiac hypertrophy and ischemia/reperfusion injury respectively.

Altogether, the accumulating evidence suggests that mitochondrial fission and fusion are both essential for mitochondrial QC, and deregulation of either process will result in cardiomyocyte dysfunction, cardiac remodeling and even cell death. Impaired mitochondrial fission causes dilated cardiomyopathy, while impaired mitochondrial fusion causes hypertrophy (Papanicolaou et al., 2011; Ikeda et al., 2015b; Song et al., 2015b; Hu et al., 2019). Nevertheless, the mechanisms underlying the associations between mitochondrial fission or fusion and pathological conditions are unclear. Additionally, there are some conflicting observations in mitophagy and mitochondrial dynamics, which should be further confirmed in future studies (Figure 3).

## Mitophagy in Cardiovascular Disease (CVD)

### PINK1/Parkin-Dependent Mitophagy

Parkin-dependent mitophagy has been reported to protect the heart from high-fat diet-induced cardiac hypertrophy, diastolic dysfunction, and lipid accumulation in mice (Tong et al., 2019). Evidence suggests that decreased PINK1/Parkin-dependent mitophagy is associated with development of dystrophic cardiomyopathy (Kang et al., 2018). Recently, it has been reported that PINK1/Parkin-dependent mitophagy is regulated by several factors (Sun et al., 2014; Tahrir et al., 2017; Yu et al., 2017; Li G. et al., 2018; Pickles et al., 2018; Thai et al., 2018; Xiong et al., 2018; Ren J. et al., 2019; Shang et al., 2019). PTEN is a PIP3 phosphatase that generates phosphatidylinositol (4,5) bisphosphate (PIP2), which is involved in multiple biological processes. PTEN was reported to bind Parkin via a form of the membrane binding helix in N-terminus and enhance the recruitment of Parkin onto mitochondria, leading to the removal of damaged mitochondria and maintenance of cardiomyocyte homeostasis (Li G. et al., 2018). In addition, mitophagy in cardiomyocytes is regulated by mammalian Ste20-like kinase 1 (Mst1), which rapidly increases in cardiomyocytes upon LPS treatment or diabetic stress. Mst1 depletion attenuates LPS-induced cardiomyocyte death or diabetic cardiomyopathy and improves cardiac function via promoting Parkin-dependent mitophagy (Shang et al., 2019). Further evidence reveals that the inhibition of mitophagy by Mst1 is associated with SIRT3, whereas depletion of SIRT3 restores cardiac function when Mst1 is overexpressed in diabetic cardiomyopathy (Wang S. et al., 2019). However, depletion of SIRT3 aggravates impaired mitophagy in diabetes mellitus-associated cardiac dysfunction, accompanied by decreased deacetylation of FOXO3A and expression of Parkin (Yu et al., 2017).

A recent study revealed that adenine nucleotide translocator (ANT) complex drives mitophagy independent of its nucleotide translocase catalytic activity. ANT interacts with TIM23 and suppresses TIM23-mediated protein translocation, which results in the stabilization of PINK1 and subsequent mitophagy. In mouse heart, the lack of ANT causes the accumulation of mitochondria and abnormal mitochondrial morphology with swelling structure and aberrant cristae, which is associated with cardiac hypertrophy and reduced contractile function. Interestingly, this team also found a patient who suffered from homozygous loss of function mutations in ANT1, developed severe heart failure and the biopsy indicated this patient’s cardiomyocytes had abnormal mitochondrial homeostasis (Hoshino et al., 2019).

**FIGURE 4:**
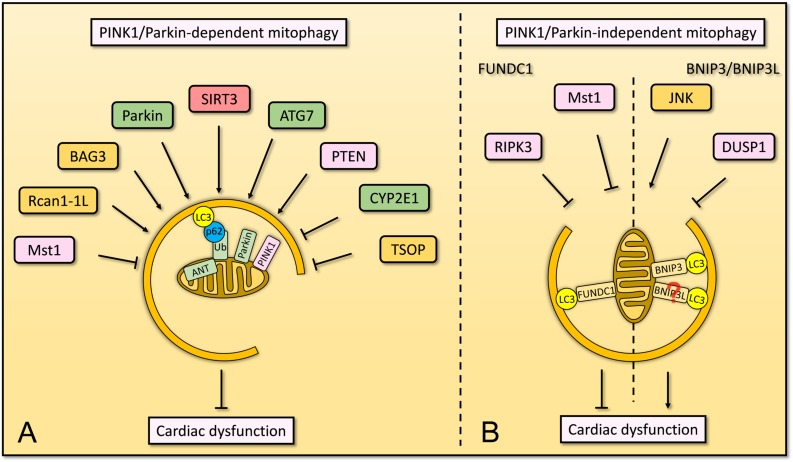
Overview of mitophagy that involves in cardiac dysfunction. **(A)** In cardiomyocytes, PINK1/Parkin-dependent mitophagy is regulated by several factors. ANT localizes on the IMM, maintains the stabilization of PINK1 and subsequent mitophagy. SIRT3 plays an important role in mitophagy to suppress diabetes mellitus-associated cardiac dysfunction. Parkin and ATG7 are essential to mitophagy for homeostasis of cardiomyocytes. PTEN regulates mitophagy and maintains mitochondrial quality control as well as cardiac homeostasis. BAG3 and Rcan1-1L are involved in mitophagy for cardiomyocyte function, whereas CYP2E1 and TSOP negatively regulate mitophagy in cardiomyocytes. Mst1, serves as pro-apoptotic kinase, inhibits PINK1/Parkin-dependent mitophagy and promotes cardiac dysfunction. **(B)** Cardiac function is also regulated by PINK1/Parkin-independent mitophagy, including FUNDC1-dependent and BNIP3-dependent mitophagy. FUNDC1-dependent mitophagy restores cardiac function by Mst1 and RIPK3, whereas BNIP3-dependent mitophagy induces cardiac dysfunction which is promoted by JNK pathway but is inhibited by DUSP1. The knowledge linking BNIP3L to cardiomyocytes via mitophagy remains limited.

Several lines of evidence suggested that normal PINK1/Parkin-dependent mitophagy is required for the maintenance of cardiac function (Figure 4A). However, a few proteins were known to regulate mitophagy in this manner and more definitive proofs of their clear mechanisms are needed.

### PINK1/Parkin-Independent Mitophagy

It has been mentioned above that the activity of FUNDC1 in mitophagy is regulated by PTMs (Liu et al., 2012; Wu et al., 2014). FUNDC1, via binding to the ER-resident inositol 1,4,5-trisphosphate type 2 receptor (IP_3_R2), modulates Ca^2+^ release from the ER at mitochondria-associated ER membranes (MAMs) to maintain mitochondrial function and homeostasis, which is essential for cardiac function, and to protect the heart from failure (Wu et al., 2017). Apart from regulating SIRT3, Mst1 represses FUNDC1 expression and FUNDC1-induced mitophagy in cardiac I/R injury via the ERK-CREB pathway. Depletion of Mst1 sustains mitochondrial homeostasis and protects cardiomyocytes (Yu W. et al., 2019). In contrast, Receptor-interacting serine/threonine-protein kinase 3 (RIPK3) induces mitochondrial apoptosis in cardiac I/R injury by inhibiting FUNDC1-mediated mitophagy (Quinsay et al., 2010).

The interaction between BNIP3 and LC3 is positively regulated by phosphorylation of Ser17 and Ser24 in BNIP3 (Liu et al., 2014). In cardiomyocytes isolated from rat heart, overexpression of BNIP3 induces extensive mitophagy, which is independent of cytosolic Ca^2+^ level, ROS generation and mPTP opening (Quinsay et al., 2010). The expression of BNIP3 increases in cardiomyocytes under PE or calcium stress *in vitro* or in response to pressure overload *in vivo*. By 3-methyladenine treatment and gene ablation assay, JNK signaling was proved to regulate BNIP3 expression via the transcription factor FOXO3a, thus protecting cardiomyocytes from cardiac remodeling in heart failure (Chaanine et al., 2012). JNK signaling is also regulated by Dual-specificity protein phosphatase1 (DUSP1) to alleviate cardiac I/R injury. This effect involves suppression of MFF-mediated mitochondrial fission and BNIP3-dependent mitophagy *in vivo* (Jin et al., 2018).

Structurally similar to BNIP3, BNIP3L, is also a receptor for mitophagy. BNIP3L interacts with LC3 or GABARAP by its WXXL motif in the N-terminal region to remove the excess or damaged mitochondria (Kanki, 2010; Moyzis and Gustafsson, 2019). However, serving as pro-apoptotic factors, BNIP3L is involved in cardiomyocyte apoptosis. Evidence indicates increased BNIP3L promotes cardiomyocyte apoptosis and causes cardiomyopathy with LV dilation and represses systolic function, whereas reduced BNIP3L protects against apoptotic cardiomyopathy (Lynch et al., 2002; Diwan et al., 2008). Although BNIP3L is widely studied in cardiomyocytes acting as a pro-apoptotic factor, definitive proofs linking cardiomyocytes to the role of BNIP3L in mitophagy are not available.

In summary, evidence from several studies suggests that mitophagy in cardiomyocytes may be a critical pathway for mitochondrial QC. Mitophagy receptors such as FUNDC1, BNIP3, BNIP3L, etc., play essential roles in maintenance of mitochondrial and cellular homeostasis (Figure 4B). For a number of these receptors, their mechanism of action remains unclear and needs to be further investigated. In addition, based on emerging knowledge about dedicated mitophagy receptors, some novel pharmacological agents have been proposed to prevent CVD. These agents include 6-Gingerol, berbamine and melatonin (Zhou L. et al., 2017; Ren Q. et al., 2019).

## Mitochondrial-Derived Vesicles (MDVS) in Cardiovascular Disease (CVD)

Although few researchers have studied the association between MDVs and cardiomyocytes, Cadete et al. (2016) revealed that MDVs maintain the homeostasis of mitochondria in cardiomyocytes in response to acute stress induced by doxorubicin. They also suggested that MDV formation serves as baseline housekeeping mechanism and a first line of defense against stress in the cardiac system (Cadete et al., 2016).

This preliminary evidence suggests that there may be a link between MDVs and cardiomyocyte function. However, much more work is needed to investigate the potential association between MDVs and CVD. The specific mechanisms underlying generation and maturation of MDVs also require further study. In particular, it will be important to identify the kinds of proteins that are involved in generating the selective asymmetry of MDVs.

## Pharmacological and Therapeutic Targets Based on Mitochondrial Quality Control Systems

Recently, many studies have focused on therapies targeted to mitochondrial QC including PTMs on mitochondrial proteins, mitochondrial dynamics, mitophagy, and MDVs. Melatonin treatment was reported to maintain myocardial function and cardiomyocyte viability in the context of I/R injury *in vivo*. The effect was related to OPA1-dependent maintenance of mitochondrial fusion and mitophagy, and occurred through activation of AMPK by melatonin (Zhang et al., 2019). By inhibiting Mst1, melatonin also increases the expression of Parkin and thus restores Parkin-mediated mitophagy to protect the heart from diabetic cardiomyopathy (Wang S. et al., 2018). 7,8-dihydroxyflavone (7,8-DHF), a mimetic of Brain-derived neurotrophic factor (BDNF), also attenuates cardiomyocyte death and improves cardiac function in cardiac ischemic injury via repression of the OMA1-dependent proteolytic cleavage of L-OPA1 (Wang Z. et al., 2019). In addition to mitochondrial fusion, fission can also be targeted to improve cardiomyocyte function under pathological conditions. For example, during I/R injury, treatment with Mdivi-1, improves cardiac function, attenuates arrhythmia and reduces infarct size by maintaining mitochondrial function in cardiomyocytes, resulting in suppression of cellular death. Mdivi-1 is more efficient when administered prior to ischemia, but less effective at the onset of reperfusion (Cooper and Eguchi, 2018). Through reducing DRP1 expression levels, Mdivi-1, suppresses cell death, inhibited mPTP opening, and protected heart from I/R injury (Piantadosi et al., 2008; Ong et al., 2010). The pretreatment of FK506 and therapeutic hypothermia (30°C) also preserved cardiac function via preventing DRP1-S637 dephosphorylation (Ong et al., 2010). Also, treatment with P110, which inhibits the DRP1/FIS1 interaction, improves mitochondrial homeostasis in cardiomyocytes and promotes the removal of damaged mitochondria in Huntington’s disease models (Joshi et al., 2019). A traditional Chinese medicine, Shenmai, induces mitophagy, regulates mitochondrial dynamics, improves intracellular Ca^2+^ homeostasis and mitochondrial potential to attenuate H/R injury *in vitro* and to prevent Left Anterior Descending Coronary Artery Ligation (LAD)-induced cardiac dysfunction *in vivo* (Yu J. et al., 2019).

As mentioned above, by targeting to autophagy receptors on mitochondria, or to regulators of mitochondrial function, several novel pharmacological therapies play a role in cardiovascular system. Through phosphorylating PI3K, AKT, mTOR and further suppressing BNIP3, 6-Gingerol attenuates hypoxia-induced cardiomyocyte injury (Ren Q. et al., 2019). Melatonin has also been identified to suppress platelet activation in cardiac I/R injury by restoring PPARγ and thus blocking FUNDC1-dependent mitophagy, which leads to reduction of platelet activity and improved cardiac outcome (Zhou L. et al., 2017). In addition to pharmacological therapies, several physical therapies are also efficient in preventing cardiac disease. For example, in aged mice after myocardial infarction, short-duration swimming exercise (15 min) was more effective than long-duration swimming (60 min) at regulating mitochondrial dynamics and mitophagy. Cardiac function was improved and the expression of SIRT3 was promoted. However, depletion of SIRT3 in this study impairs mitochondrial dynamics, promotes ROS generation and release, and causes cell death (Zhao et al., 2018).

Thus, by targeting proteins involved in mitochondrial QC, both pharmacological and mechanical therapies can maintain mitochondrial homeostasis in cardiomyocytes. As described earlier in this review, there are several targets of mitochondrial QC in cardiomyocytes, and therefore more drugs or treatments are expected to be proposed and further developed for clinical use.

## Future Perspectives

Following different extracellular stresses, perturbations in mitochondrial homeostasis result in abnormal mitochondrial morphology, dysfunctional oxidative phosphorylation, leakage of components from the mitochondrial matrix, and activation of mitochondrion-dependent cellular signaling. Under physiological conditions, PTMs on mitochondrial proteins maintain the balance between mitochondrial fusion and fission as well as executing mitophagy to remove aged mitochondria for normal cellular homeostasis. Under pathological conditions, such as I/R injury or exposure to cardiotoxic agents, PTMs (SUMOylation, phosphorylation, ubiquitination) on critical mitochondrial proteins trigger changes in mitochondrial dynamics and mitophagy to rescue cellular homeostasis. Currently, many studies are focusing on the effect of mitochondrial QC in cardiomyocytes, including PTMs on mitochondrial proteins, mitochondrial dynamics, MDVs and mitophagy, trying to connect some known molecular mechanisms to cardiomyocytes and revealing the cardiac-specific mechanism of mitochondrial QC. The mechanisms by which mitochondrial QC protects cardiomyocytes against cardiac disease are becoming increasingly clear, and consequently more and more potential targets for clinical therapies are emerging. Furthermore, we should pay attention to that mitochondrial dynamics and network is highly dynamic and complex. Although accumulating tools or techniques are studied for real-time tracing of mitochondria *in vitro*, precise and effective methods remain in need of that *in vivo* (Guo et al., 2018).

Therefore, key goals for the future include: investigating more characterization of PTMs on mitochondrial proteins; developing more means to trace mitochondrial dynamics and network; identifying more solid and direct associations between protein targets of mitochondrial QC and specific CVDs; developing more efficient pharmacological or physical therapies for protecting the heart; and considering how to transfer basic research results into clinical practice.

## Author Contributions

HF and DF conceived and wrote the manuscript. All authors commented and revised the manuscript.

## Conflict of Interest

The authors declare that the research was conducted in the absence of any commercial or financial relationships that could be construed as a potential conflict of interest.

## Abbreviations

7,8-DHF: 7,8-dihydroxyflavone
AKT: RAC-alpha serine/threonine-protein kinase
AMPK: adenosine monophosphate-activated protein kinase
ANT: adenine nucleotide translocator
APC/CCdh1: Anaphase-promoting complex/cyclosome and its coactivator Cdh1
ATO: Arsenic trioxide
BDNF: brain-derived neurotrophic factor
BNIP3: BCL2/adenovirus E1B 19 kDa protein-interacting protein 3
BNIP3L/NIX: BCL2/adenovirus E1B 19 kDa protein-interacting protein 3-like
CaMK1 α: Ca ^2 +^ /calmodulin-dependent protein kinase-1 α
CDK1: Cyclin-dependent kinase 1
CREB: cyclic AMP-responsive element-binding protein
CVD: cardiovascular disease
DRP1: dynamic relative-protein 1
DUSP1: Dual-specificity protein phosphatase1
E1: activating enzymes
E2: conjugating enzymes
E3: ligase enzymes
ER: endoplasmic reticulum
ERK: extracellular signal-regulated kinase
FIS1: mitochondrial fission 1 protein
FOXO: Forkhead box protein O
FUNDC1: FUN14 domain-containing protein 1
GNAT: Guanine nucleotide-binding protein G(t) subunit alpha
GqPCR: Gq protein-coupled receptor
GSK: glycogen synthase kinase
H/R: Hypoxia/reoxygenation
HATs: histone acetyltransferases
HDACs: histone deacetylases
HO1: Heme oxygenase 1
I/R: Ischemia/reperfusion
IMM: inner mitochondrial membrane
IP_3_R2: ER-resident inositol 1,4,5-trisphosphate type 2
KATs: lysine acetyltransferases
KDACs: lysine deacetylases
LAD: Left Anterior Descending Coronary Artery Ligation
LC3: Microtubule-associated proteins 1A/1B light chain 3
LIR: LC3 interaction region
MAMs: mitochondria-associated ER membranes
MAPL: mitochondrial-anchored protein ligase
MARCH: Membrane-associated RING-CH
MCL1: Induced myeloid leukemia cell differentiation protein Mcl-1 homolog
MFF: mitochondrial fission factor
MFN: Mitofusin
MiD49: Mitochondrial dynamics protein of 49 kDa homolog
MID51: Mitochondrial dynamics protein of 51 kDa homolog
Miro2: Mitochondrial Rho GTPase 2
MnSOD: manganese superoxide dismutase
mPTP: mitochondrial permeability transition pore
Mst1: mammalian Ste20-like kinase 1
mTOR: Mammalian target of rapamycin
NMNAT: nicotinamide mononucleotide adenylyltransferase
NR4A1: nuclear receptor subfamily 4 group A member 1
NRF1: Endoplasmic reticulum membrane sensor NFE2L1
NRF2: Nuclear factor erythroid 2-related factor 2
OGD: Oxygen/glucose deprivation
OMM: Outer mitochondrial membrane
OPA1: Optic atrophy protein 1 homolog
p300/CBP: CREB-binding protein
PARL, Presenilins-associated rhomboid-like protein: mitochondrial
PGC1: the transcriptional coregulator peroxisome proliferator-activated receptor γ coactivator 1
PI3K: Phosphoinositide 3-kinase
PINK: PTEN-induced putative kinase protein
PIP2: phosphatidylinositol (4,5) bisphosphate
PKA: Protein kinase A
PKD: Protein kinase D
PK δ: protein kinase C δ
PPAR α: Peroxisome proliferator-activated receptor α
PTEN: Phosphatidylinositol 3,4,5-trisphosphate 3-phosphatase and dual-specificity protein phosphatase
PTM: post-translational modification
Q73: long polyglutamine repeat
QC: quality control
RIPK3: Receptor-interacting serine/threonine-protein kinase 3
ROCK1: Rho-associated coiled-coil-containing protein kinase 1
ROS: reactive oxidative species
S3: Diterpenoid derivative 15-oxospiramilactone
SENP: Sentrin/small ubiquitin-like modifier-specific protease
SIRT: NAD-dependent protein deacetylase sirtuin-1
SOD: Superoxide dismutase
SUMO: Small Ubiquitin-like Modifier
UCP1: Mitochondrial brown fat uncoupling protein 1
ULK1: Serine/threonine-protein kinase ULK1
UPS: Ubiquitination Proteasome System
VDAC1: Voltage-dependent anion-selective channel protein 1.
